# An immortalized adult human erythroid line facilitates sustainable and scalable generation of functional red cells

**DOI:** 10.1038/ncomms14750

**Published:** 2017-03-14

**Authors:** Kongtana Trakarnsanga, Rebecca E. Griffiths, Marieangela C. Wilson, Allison Blair, Timothy J. Satchwell, Marjolein Meinders, Nicola Cogan, Sabine Kupzig, Ryo Kurita, Yukio Nakamura, Ashley M. Toye, David J. Anstee, Jan Frayne

**Affiliations:** 1 School of Biochemistry, University of Bristol, Bristol BS8 1TD, UK; 2 Department of Biochemistry, Faculty of Medicine Siriraj Hospital, Mahidol University, Bangkok 10700, Thailand; 3 Bristol Institute for Transfusion Sciences, National Health Service Blood and Transplant (NHSBT), Bristol BS34 7QH, UK; 4 NIHR Blood and Transplant Research Unit, University of Bristol, Bristol BS8 1TD, UK; 5 School of Cellular and Molecular Medicine, University of Bristol, Bristol BS8 1TD, UK; 6 Department of Research and Development, Central Blood Institute, Japanese Red Cross Society, Tokyo 135-8521, Japan; 7 Cell Engineering Division, RIKEN BioResource Center, Ibaraki 305-0074, Japan

## Abstract

With increasing worldwide demand for safe blood, there is much interest in generating red blood cells *in vitro* as an alternative clinical product. However, available methods for *in vitro* generation of red cells from adult and cord blood progenitors do not yet provide a sustainable supply, and current systems using pluripotent stem cells as progenitors do not generate viable red cells. We have taken an alternative approach, immortalizing early adult erythroblasts generating a stable line, which provides a continuous supply of red cells. The immortalized cells differentiate efficiently into mature, functional reticulocytes that can be isolated by filtration. Extensive characterization has not revealed any differences between these reticulocytes and *in vitro*-cultured adult reticulocytes functionally or at the molecular level, and importantly no aberrant protein expression. We demonstrate a feasible approach to the manufacture of red cells for clinical use from *in vitro* culture.

## Abstract (web summary)

The generation of a sustainable supply of erythroid progenitors is essential for the reliable production of an *in vitro* derived red blood cell clinical product. Here the authors immortalize early human erythroblasts to generate the first cell line capable of differentiation into functional adult reticulocytes.

Blood shortage is an important healthcare problem globally, anticipated to become more problematic as people live longer and donor numbers dwindle. There is therefore need for an alternative red cell product. Cultured red blood cells provide such an alternative and have potential advantages over donor blood, such as a reduced risk of infectious disease transmission, and as the cells are all nascent, the volume and number of transfusions administered to patients requiring regular transfusions (sickle cell disease, thalassaemia myelodysplasia, certain cancers) could be reduced, ameliorating the consequences of organ damage from iron overload.

Various sources of stem cells, adult peripheral blood (PB), umbilical cord blood (CB) and pluripotent^1^,^2^,^3^,^4^,^5^,^6^, have been used as progenitors for *in vitro* erythroid culture systems, all differentiating along the erythroid pathway. However, PB progenitors have a limited proliferative capacity^7^, which restricts the number of red cells that can be obtained, greatly impacting the economic viability of producing therapeutic quantities of red cells from this source. CB progenitors have a greater expansion capacity than PB progenitors, but the number of cells generated is still limited and the cells have a fetal, rather than adult, phenotype. Pluripotent stem cells (PSCs) provide a potentially unlimited progenitor source; however, there are substantial hurdles to overcome before these cells can be considered for manufacture of red cells, not least the small number of erythroid progenitors generated to date and severely impaired enucleation of the resultant erythroid cells.

Another strategy is to generate immortalized adult erythroid progenitor cell lines. Such lines are capable of providing an unlimited supply of red cells and need only minimal culture to generate the final product. This process avoids the complex and lengthy differentiation required for PSCs and the need for repeat donations of PB and cord progenitors. The first therapeutic use of a cultured red blood cell product will likely be for patients with rare blood group phenotypes because suitable conventional red cell products are difficult to source. Immortalized lines could be generated with selected blood group phenotypes to meet the needs of such patients.

Most available continuous cell lines with erythroid characteristics are derived from patients with myelogenous leukaemia or erythroleukaemia and do not represent ‘normal' erythroid cells. To date, there are very few reports in the literature on attempts to generate immortalized lines of normal human erythroid cells. Lines have been generated using erythroid cells differentiated from human induced PSCs (HiDEP^8^), CB progenitors (HUDEP^8^; iE^9^) and embryonic stem cells^10^. However, all express fetal or embryonic globin and have terminal differentiation defects. There are no reports describing the generation of immortalized lines from normal adult human erythroid cells, although such cells would be extremely valuable.

In this study we generate the first human immortalized adult erythroid line (Bristol Erythroid Line Adult; BEL-A), which provides a sustainable supply of erythroid cells. It is the first erythroid line to fully recapitulate normal erythropoiesis, enucleating to generate mature reticulocytes, characterization of which revealed no differences functionally or at the molecular level to *in vitro*-cultured adult reticulocytes, and a preliminary test demonstrates similar survival rates to adult donor RBCs *in vivo*. The technique is robust and reproducible, with multiple additional lines successfully generated. In addition to facilitating the development of novel therapeutic products, BEL-A is easily transduced and a superior research tool for the study of erythropoiesis and RBC disease.

## Results

### Generation of immortalized adult erythroid line

**Figure 1:**
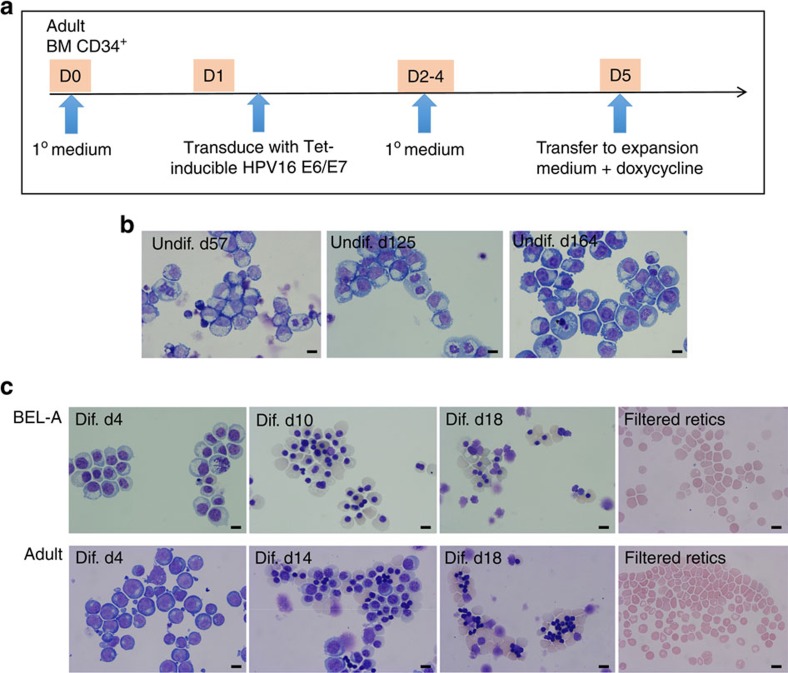
Generation of immortalized adult erythroid cell line. Human adult bone marrow CD34^+^ cells were recovered from frozen primary erythroid culture medium for 24 h, before transduction with Tet-inducible HPV16 E6/E7 construct. Cells were transferred to StemSpan expansion medium with doxycycline on day 5 in culture and maintained in expansion medium thereafter. (**a**) Schematic of experimental approach. (**b**) Representative cytospins illustrating similar morphology of cells maintained proliferating in continuous culture on days 57, 125 and 164. (**c**) Representative cytospin images showing morphology of cells on days 4, 10 and 18 following transfer to erythroid differentiation medium and of reticulocytes isolated by filtration. Cytospins of erythroid cells differentiated from adult peripheral blood CD34^+^ at comparative stages of differentiation are included for comparison. Cells were stained with May–Grunwald Giemsa reagent and analysed by light microscopy. Scale bars 10 μm.

We set out to create immortalized adult erythroid lines, utilizing a Tet-inducible HPV16-E6/E7 expression system^8^. Adult bone marrow CD34^+^ cells were transduced with the HPV16-E6/E7 construct and maintained in primary medium of our erythroid culture system^3^ for 4 days. On day 5 cells were transferred to expansion medium containing doxycycline to induce expression of E6 and E7, and were maintained in this medium thereafter. Figure 1a shows a schematic of the protocol. The cells proliferated continuously and after 190 days, well beyond the Hayflick limit^11^, were frozen for storage. Samples were also frozen at regular intervals throughout this time period. All samples re-established efficiently in culture following freeze thawing. The line was named BEL-A for Bristol Erythroid Line Adult. The mean doubling time of the cells after day 100 in continuous culture was 20 h. Morphological analysis of the immortalized cells showed that they are pro- to early basophilic erythroblasts. There was no change in morphology over time (Fig. 1b).

BEL-A cells after 100 days in continuous culture were transferred to primary erythroid culture medium containing doxycycline for 6 days, and then to tertiary medium^3^ without doxycycline to induce differentiation to mature erythroblasts (orthochromatic normoblasts) and reticulocytes (Fig. 1c). Comparison with erythroid cells differentiated from adult PB CD34^+^ progenitor cells showed similar morphological progression down the erythroid pathway (Fig. 1c). BEL-A cells transferred to differentiation medium at regular time points post day 100 in culture showed unchanged ability to differentiate. The expansion profile of such cells was also consistent (Supplementary Fig. 1). Cells enucleated up to 30%, with stable reticulocytes isolated by filtration using a standard leukocyte reduction filter^3^ (Fig. 1c). Cultured adult reticulocytes were 9.8±1 μm (mean±s.d., *n*=100) in diameter and BEL-A reticulocytes 9.9±1 μm (mean±s.d., *n*=100). We confirmed that expression of HPV16 E6 and E7 was lost from BEL-A cells following removal of doxycycline (Supplementary Fig. 2).

### Characterization of the BEL-A line

Morphologically, erythroid cells and reticulocytes differentiated from the BEL-A line appear identical to those derived from adult PB CD34^+^ cultures. We next extensively characterized the properties of BEL-A cells in comparison with these adult erythroid cells. Extensive characterization of *in vitro* adult reticulocytes has been carried out previously and reveals no substantive differences compared to endogenous cells^3^,^12^,^13^. Furthermore, the cultured reticulocytes survive and mature *in vivo*^12^,^14^,^15^.

Erythroid cells undergo distinct changes in their membrane protein expression profile during erythropoiesis, with some proteins increasing and others decreasing in level as the cells differentiate^16^,^17^,^18^. To determine if BEL-A cells exhibit a normal expression profile of membrane proteins during differentiation we compared protein levels during erythropoiesis with that of erythroid cells differentiated from adult PB CD34^+^ cells by flow cytometry, using antibodies to a panel of established RBC antigens. The dynamic expression pattern was equivalent for both populations of cells (Supplementary Fig. 3a,b). We also compared the abundance of glycophorin A (GPA) and band 3 in BEL-A reticulocytes with that of *in vitro* adult reticulocytes and endogenous RBCs (Supplementary Fig. 3c). The level of both proteins was equivalent in the reticulocyte populations, but lower in RBCs, as expected. The data also confirmed that the reticulocytes are larger than RBCs. Reticulocytes loose volume and surface area via exosomes and vesicles^3^,^19^,^20^, particularly as they mature in the circulation. GPA and band 3 have been shown to be present on such vesicles^3^,^19^, thus reducing abundance on the mature RBC.

Serological analysis confirmed that the blood group of the BEL-A reticulocytes corresponds to that of the bone marrow CD34^+^ cell donor from which they were created. Importantly, the BEL-A reticulocytes do not have T or Tn antigen exposure demonstrating normal *O*-glycosylation.

**Figure 2:**
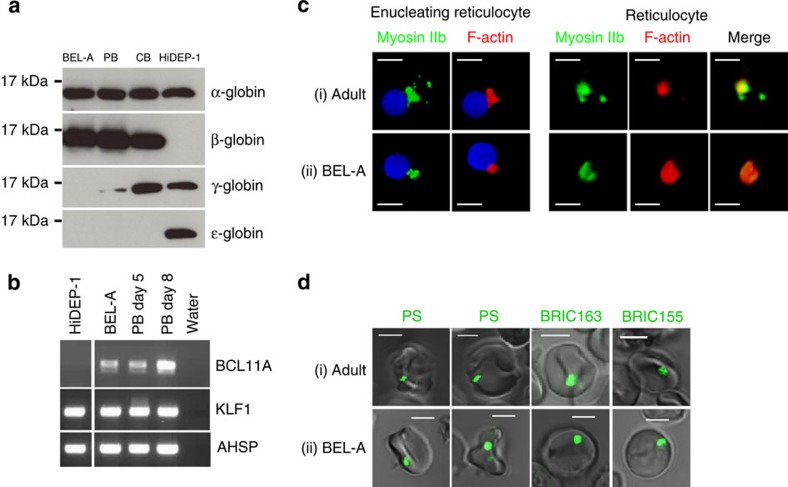
Characterization of BEL-A line. (**a**) Western blots of late stage BEL-A, HiDEP-1 and erythroid cells differentiated from adult PB and cord blood CD34^+^ cells (at days 18, 12, 19 and 18 in culture, respectively) incubated with antibodies to α-, β-, γ- and ɛ-globin. (**b**) Expression of BCL11A and KLF1 transcripts in expanding BEL-A and HiDEP-1 cells and erythroid cells differentiated from adult PB CD34^+^ cells at days 5 and 8 in culture by RT-PCR. Transcripts for AHSP were included as a positive control. (**c**) Erythroid cells differentiated from adult peripheral blood CD34^+^ cells (i) and BEL-A cells (ii) were fixed, permeabilized and dual stained for F-actin (red) and myosin IIb (green). Single cells showing F-actin and myosin IIb localization in an enucleating cell, and in a reticulocyte are shown in three dimension. (**d**) Filtered adult reticulocytes (i) and BEL-A reticulocytes (ii) were live imaged after staining for phosphatidylserine (PS), glycophorin A intracellular domain (BRIC163) and band 3 intracellular domain (BRIC155) (all green). Scale bars 5 μm.

Before differentiation centrifuged BEL-A cells produced a pink pellet, but a red pellet following differentiation, indicating haemoglobin synthesis. We compared the globin expression profile of BEL-A cells to that of erythroid cells differentiated from adult PB CD34^+^ at the same stage of differentiation. Erythroid cells differentiated from CB CD34^+^ cells were included as a positive control for *γ-globin* expression, as well as cells from one of the previously generated immortalized erythroid lines, HiDEP-1 (ref. 8). BEL-A cells synthesized exclusively β-globin at the level detected in adult erythroid cells; densitometry of the globin bands on western blot gave a ratio of BELA:PB β-globin of 1.04 (Fig. 2a). In contrast, HiDEP-1 cells expressed predominantly ɛ- with some γ-globin, as we have shown previously^21^. These data were corroborated by HPLC globin typing whereby erythroid cells at day 20 from an adult culture contained 94.9% HbA, 3.1% HbA2 and 2% HbF, BEL-A cells at day 12 in culture contained 99.3% HbA and 0.7% HbF and HiDEP-1 cells contained 3.8% HbA, 40.3% HbF and 55.9% HbE. We compared the level of transcription factors KLF1 and BCL11A in these cells since both are required for the switch to and expression of β-globin^21^. The level of *KLF1* transcripts in BEL-A and HiDEP-1 cells was similar to that detected in adult erythroid cells. However, whereas BEL-A cells expressed normal adult levels of BCL11A, no transcripts were detected in HiDEP-1 cells, consistent with their failure to express *β-globin* (Fig. 2b). A number of other key erythroid transcription factors in BEL-A cells were expressed equivalently to adult erythroid cells at a similar stage of differentiation (Supplementary Fig. 4).

A major barrier to the use of PSCs for the manufacture of red cells is the failure of erythroblasts generated to enucleate correctly. F-actin and myosin are known to form a contractile actin ring proximal to the site of nuclear extrusion^22^,^23^, with myosin IIb implicated in enucleation^24^. We show that Myosin IIb is a component of the F-actin core, with co-localization apparent at the point of nuclear extrusion and persisting in early reticulocytes generated from PB derived CD34^+^ cells (Fig. 2c). This apparent association between F-actin and myosin IIb is also seen in BEL-A cells (Fig. 2c). In contrast, in HiDEP-1 cells, which are derived from induced PSCs, enucleated cells are fragile and morphologically abnormal with an irregular plasma membrane and lack a myosin and F-actin core (Supplementary Fig. 5).

The vesicles extruded from reticulocytes during maturation to an erythrocyte have been shown to be large, intact, inside-out phosphatidylserine-exposed autophagic vesicles^19^. Live cell imaging of filtered BEL-A reticulocytes confirmed the presence of such vesicles at the cell surface (Fig. 2d), further demonstrating their comparability with adult cultured reticulocytes.

### Comparing the proteome of BEL-A and adult reticulocytes

Comparative proteomics of BEL-A reticulocytes (three replicates) and reticulocytes isolated from cultures of PB CD34^+^ cells from three separate individuals were performed. No proteins detected were unique to the BEL-A or *in vitro-*cultured adult reticulocytes. Of the quantified proteins, 1964 were common to all BEL-A and adult replicates (Supplementary Data 1). Of these, 19 (<1%) were more abundant in the BEL-A reticulocytes and 191 (9.7%) in the adult reticulocytes (Supplementary Data 2a,b). Notably, 31% of the latter were ribosome proteins and 15% mitochondrial proteins, known to be progressively lost during reticulocyte maturation (reviewed in Ney (2011)^20^). Upon further analysis, and cross reference with our data mapping the change in proteome between *in vitro*-cultured adult reticulocytes and RBCs, it was apparent that the differences were due to the stage of reticulocyte maturation, with a shift in the relative distribution between the populations: heterogeniety in maturation of reticulocyte populations having previously been reported^25^. The data files were also searched against the Uniprot human papillomavirus type 16 database, with no viral proteins detected in the reticulocyte samples.

### Functional analysis of BEL-A cells

We also addressed BEL-A red cell function. Normal deformability of the BEL-A reticulocytes is essential for clinical use. Comparison of BEL-A and normal adult reticulocytes using an Automated Rheoscope Cell Analyser^26^ showed comparable deformability indexes (Supplementary Fig. 6). The cells also bound and released oxygen in a manner comparable to normal adult erythroid cells (Supplementary Fig. 7).

### *In vivo* survival of BEL-A reticulocytes

To evaluate survival and maturation of BEL-A reticulocytes *in vivo*, macrophage-depleted NSG mice^15^ were transfused with BEL-A cells or adult donor RBCs. Overall, there were no difference in the survival rate of BEL-A and donor RBC detected in the murine circulation over the evaluation period (Supplementary Fig. 8a,b). The diameter of BEL-A cells decreased between 10 min and 24 h post transfusion (Supplementary Fig. 8c), with cells at 24 h exhibiting mature erythrocyte morphology (Supplementary Fig. 8d).

### Large-scale culture of BEL-A cells

To evaluate the feasibility of transfer to larger scale production, BEL-A cells grown under static culture conditions to 250 ml were transferred to 1.5 l spinner flasks (Supplementary Fig. 9). The doubling rate of the cells was unchanged and they differentiated normally on transfer to differentiation medium, demonstrating that the cells will grow in stirred vessels as well as in static culture, which is important for development of future scale-up procedures and demonstrates a potential pathway to therapeutic productivity. To date, optimal protocols for such large-scale culture and bioreactor vessels for the manufacture of red cells at a clinical scale have not been reported, but the same hurdles will apply for erythroid cultures using all stem cell sources as progenitors. The feasibility of developing such a system, along with associated projected costs, has been extensively reviewed^27^,^28^,^29^. Alongside further work required to optimize enucleation, reticulocyte stability in culture and efficient methods for reticulocyte purification required for escalation of culture yields, one of the next steps for our procedure is transfer to GMP conditions. We do not envisage significant hurdles with such transfer, as, for example Timmins *et al*.^30^ using a similar erythroid differentiation system found no effect on culture performance when fully animal component-free media was substituted.

### Amenability of BEL-A cells to genetic manipulation

**Figure 3:**
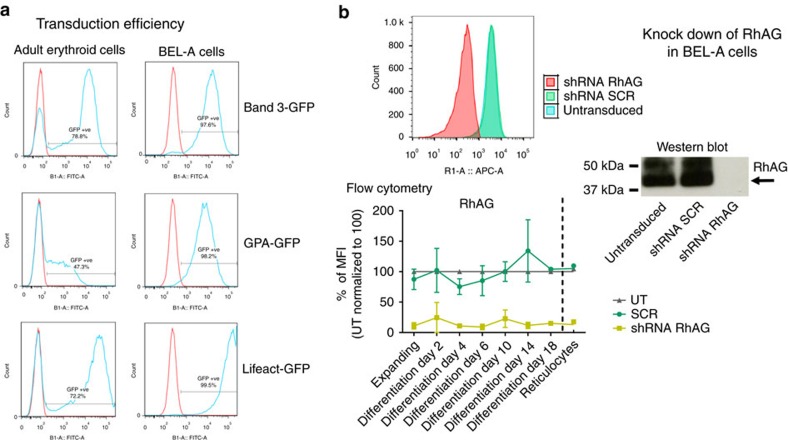
Lentiviral manipulation of BEL-A line protein expression. (**a**) BEL-A cells and pro-erythroblasts differentiated from adult peripheral blood CD34^+^ cells transduced with GPA-GFP, band 3-GFP and Lifeact-GFP, using identical cell numbers and virus batches. Transduction efficiency (shown on graphs) for each gene was measured 72 h post transduction by flow cytometry using the same voltage settings for both cell types. (**b**) Levels of RhAG measured by flow cytometry and western blot following knock down of RhAG by shRNA. A non-targeting scramble shRNA (SCR) was included as a control. The trace for untransduced cells (blue) is masked by that for SCR (green). Transduced cells were expanded for 7 days before transfer to differentiation medium. Data shown are mean±s.d. *n*=2. Western blot was performed with cells at day 8 of differentiation using a RhAG polyclonal antibody.

The BEL-A line is a valuable research resource, superior to any presently available for the study of erythropoiesis and red blood cells disorders. To illustrate the utility of these cells for genetic manipulation BEL-A cells were transduced with GPA-GFP, band 3-GFP, Lifeact-GFP and RhAG shRNA. Transduction efficiency of the GFP constructs using lentivirus was extremely high (average 98%; Fig. 3a), and the tagged proteins correctly localized to the plasma membrane. Knock down of RhAG with shRNA achieved a striking reduction in the level of protein (average 96%; Fig. 3b), and this reduction was maintained during expansion of the cells, throughout differentiation and in the resultant reticulocytes, demonstrating the capacity for generating sublines that mimic rare phenotypes. Expression of the transduced genes and RhAG was maintained at the same level following freeze thawing of the cells.

## Discussion

Our immortalization technique is robust and reproducible; we have now generated multiple additional lines, which are undergoing characterization. The use of viral oncoproteins to create these lines is unlikely to pose a risk to clinical use, as their expression is lost before terminal differentiation. In addition, the resultant product is enucleated and hence tumorigenicity negated. However, custom filter systems and rigorous assay(s) to detect any remaining nucleated cells in the final product are being investigated, as would be required for erythroid cultures from all stem cell sources. Notwithstanding, any remaining nucleated cells will be orthochromatic normoblasts with condensed nuclei, which are unlikely to be viable.

The BEL-A line demonstrates the great potential of immortalized erythroid lines for manufacture of novel red cell products for clinical use and as a research tool for the study of erythropoiesis in health and disease, with exciting applications in other research fields, for example, exploring host receptors for parasite invasion. Diverse lines could be generated from individuals with rare blood group phenotypes or generated by genome-editing technologies^31^,^32^ such as CRISPR, to meet unmet clinical need. In the future cell lines produced under optimized GMP conditions could potentially provide a source of red cells for transfusion in areas of the world where blood supplies are inadequate or unsafe.

## Methods

### Generation and differentiation of immortalized line

Adult human CD34^+^ haematopoietic progenitor cells isolated from bone marrow were purchased from Stemcell Technologies Inc. (Cambridge, UK). The frozen cells (3 × 10^5^ cells) were recovered in primary medium (IMDM (Biochrom, Cambourne, UK) containing 3% (v/v) AB Serum, 10 μg ml^−1^ insulin, 3 U ml^−1^ heparin (all from Sigma-Aldrich, Poole, UK), 2% (v/v) fetal bovine serum (Hyclone, Fisher Scientific Ltd, UK), 3 U ml^−1^ EPO (Roche, Basel, Switzerland), 200 μg ml^−1^ Transferrin, 10 ng ml^−1^ stem cell factor (SCF) and 1 ng ml^−1^ IL-3 (all from R&D Systems, Minneapolis, MN, USA) of our three-stage erythroid culture system^4^ for 24 h. They were then transduced with the Lentiviral vector CSIV-TRE-HPV16-E6/E7-UbC-KT^7^ and maintained in primary medium for 4 days. On the fifth day they were transferred to expansion medium (StemSpan SFEM (Stemcell Technologies Inc.), 50 ng ml^−1^ SCF (R&D Systems), 3 U ml^−1^ EPO (Roche) and 10^−6^ M dexamethasone (Sigma-Aldrich)), along with 1 μg ml^−1^ doxycycline (Takara Bio, Saint-Germain-en-Lave, France) to induce expression of HPV-E6 and E7, and maintained in this medium thereafter at a density of 1–3 × 10^5^ cells ml^−1^ at 37 °C, 5% CO_2_. Total medium changes with fresh medium were performed 2–3 times per week.

To induce erythroid differentiation, proliferating immortalized erythroid cells were transferred to primary medium of our culture system^3^ with the addition of 1 μg ml^−1^ doxycycline and maintained for 6 days. On day 6, the cells were transferred to the tertiary medium (primary medium with 500 μg ml^−1^ Transferrin but without SCF and IL3) of our culture system. The cultured cells were counted every other day, and fresh medium was added to obtain a final concentration at 1–2 × 10^5^ cells ml^−1^ for the first 6 days and 1 × 10^6^ cells ml^−1^ thereafter. The cells were cultured at 37 °C, 5% CO_2_ for up to 18 days. Reticulocyte filtrations were performed on days 15–18 of culture. A leukocyte reduction filter (Pall Corporation, Portsmouth, UK) was pre-wet with IMDM medium (Biochrom). The cultured cell suspension was loaded into the filter followed by a large amount of HBSS (Sigma-Aldrich) until the flow through was clear. The flow through containing reticulocytes was collected and the reticulocytes harvested via centrifugation at 600 *g* for 5 min.

### Antibodies

β-Globin (37-8; SC-21757), γ-globin (51-7; SC-21756) and α-globin (D-16; SC-31110) were all from Santa Cruz Biotechnology (Santa Cruz, CA, USA) and used at 1:2,000 dilution for western blot. ɛ-Globin (ab156041) used at 1:200 dilution for western blot and myosin IIb (ab24761) used at 1:300 dilution for confocal microscopy were from Abcam (Cambridge, MA, USA). RhAG (YUDI) used at 1:1,000 dilution for western blot was made in house. GPA (BRIC 163 used at 1:100 dilution for confocal microscopy and BRIC 256 used at 14 μg ml^−1^ for flow cytometry) and band 3 (BRIC 155 used at 1:100 dilution for confocal microscopy) were from IBGRL Research Products (Bristol, UK). GPC (BRIC10), Rh (BRIC69), RhAG (LA1818), Kell (BRIC68), band 3 (BRIC 200), CD44 (BRIC222), β_1_ integrin (TS2/16) were all from IBGRL Research Products and used at 5.7 μg ml^−1^ for flow cytometry. α4 Integrin (MAX68P), used at 5.7 μg ml^−1^ for flow cytometry, was a gift from Dr T Shock, CellTech Chiroscience (Slough, UK). CD71 (MCA1148GA) and CD147 (DF1513), both used at 5.7 μg ml^−1^ for flow cytometry, were from Bio-Rad (Hemel Hempstead, UK). Wheat Germ Agglutinin (Alexa Fluor546 conjugate), F-actin probe (Alexa Fluor546 Phalloidin) and secondary Alexa Fluor antibodies, used at 1:1,000, 1:50 and 1:500 dilution, respectively, for confocal microscopy, were from Life Technologies (Paisley, UK).

### Flow cytometry

A sample of 3 × 10^5^ cells was incubated with primary antibodies in 70 μl of phosphate-buffered saline (PBS) (Sigma-Aldrich) containing 1% (v/v) bovine serum albumin (BSA) (Lorne Laboratories, Reading, Berkshire, UK) (PBSA) for 1 h with continuously mixing. The cells were washed once with PBSA and incubated with secondary antibody RPE F(ab′)_2_ goat anti-mouse IgG (Dako Cytomation, Glossop, UK) in 25 μl PBAS for 30 min with continuous mixing in the dark. The cells were washed twice with PBSA and then analysed on a FC500 flow cytometer using Kaluza software (Beckman Coulter (UK) Ltd, High Wycombe, Buckinghamshire, UK). For detection of RhAG, cells stained for 30 min at 37 °C with 5 μg ml^−1^ Hoechst 33342 (Sigma-Aldrich). Cells were incubated with extracellular antibody LA1818 against RhAG for 30 min at 4 °C, labelled with rat APC-conjugated anti-mouse IgG_1_ (Biolegend, London, UK) and analysed on a MACSQuant system system (Miltenyi Biotech Ltd, Bisley, UK). ImageJ was used to quantify bands on western blot^33^.

### Serology

Cells were tested for the expression of major blood group antigens (ABH and Rh) using gel cards (Biorad, UK). Other cell surface antigens were detected using agglutination in test tubes with normal human sera or lectins (*Arachis hypogea*, *Glycine soja*). Reagents and methods are shown in Griffiths *et al*.^3^

### Whole-cell lysate preparation

Cultured cells were washed once with cold PBS and re-suspended in solubilization buffer (20 mM Tris HCl (pH 7.5), 150 mM NaCl, 10% glycerol, 1% Triton, 0.1% SDS, containing 1 × complete protease inhibitor and 2 mM PMSF) by pipetting up and down. After incubation for 1 h on ice, the protein samples were treated with 25 U ml^−1^ Bensonase for 1 h at 4 °C and were further centrifuged at 17,000 *g* for 5 min, at 4 °C. Supernatant was collected as protein lysate. All chemicals obtained from Sigma-Aldrich.

### SDS-PAGE and western blot

Proteins were resolved by SDS-PAGE and transferred to PVDF (Millipore) by western blotting. Membranes were blocked with 10% milk powder followed by incubation with primary (dilutions above) and secondary antibodies (rabbit α-mouse immunoglobulin-HRP, rabbit α-goat immunoglobulin-HRP and swine α-rabbit immunoglobulin-HRP; all DakoCytomation) at 1:2,000 dilution. Bands were visualized using enhanced chemilunescence (G.E. Healthcare) and exposed to Hyperfilm (G.E. Healthcare). Full blots are shown in Supplementary Figs 10 and 11.

### PCR analysis

RNA (400 ng) was reverse transcribed into cDNA using SuperScript II reverse transcriptase (Invitrogen, ThermoFisher Scientific, Waltham MA, USA). Primers (Sigma-Aldrich) used were AHSP 5′-gttagacctgaaggcagatggc and 5′-gcacttccagtctcgtctgcg; BCL11A 5′-tcacgccagaggatgacgatt and 5′-tcaagtgatgtctcggtggtgg; E2F2 5′-ctcggtatgacacttcgctgg and 5′-gcacttccagtctcgtctgcg; FOG1 5′-aaggacaggaaccagaacccag and 5′-ctctgctggctccttcttca; GATA1 5′-ttcagcagcctattcctctcc and 5′-ccttggtagagatgggcagta; KLF1 5′-cgaagagctacaccaagagc and 5′-gctgtctatgggtccgtgtt; KLF3 5′-ctactccacaccattgcctgag and 5′-cacgatgaccgaagggtgattc; NFE2 5′-gagatggaactgacttggcagg and 5′-gcacttccagtctcgtctgcg; SOX6 5′-tgatggagaggatgcaatgacc and 5′-ccatttgctgccgttgtttct; HPV16 E6 5′-gcgacccagaaagttaccac and 5′-gcaacaagacatacatcgaccgg; HPV16 E7 5′-gcaaccagagacaactgatctc and 5′-tggggcacacaattcctagtg.

### Confocal microscopy

All chemicals were from Sigma-Aldrich unless otherwise stated. All washes and dilutions were performed in Buffer A (PBS pH 7.4 containing 5 mg ml^−1^ BSA (Park Scientific, Moulton Park, UK) and 1 mg ml^−1^ glucose). Cells were seeded on 0.01% (w/v) poly-L-lysine-coated coverslips and incubated for 30 min at 37 °C in 5% CO_2_. Cells were fixed in 1% formaldehyde (TAAB, Reading, UK) and permeabilized in 0.05% saponin. After permeabilization all subsequent washes and antibody dilutions were carried out in Buffer A containing 0.005% saponin. Secondary antibodies used were goat anti-mouse Alexa fluor 488-and goat anti-rabbit Alexa fluor 546-conjugated antibodies (Invitrogen) diluted in 4% (w/v) normal goat serum. Coverslips were mounted on Vectashield Mounting Medium (Vector Laboratories, Peterborough, UK). Samples were imaged at 22 °C using × 40 oil immersion lenses (magnification 101.97 μm at zoom 3.8, 1.25 NA) on a Leica DMI 6000 inverted microscope with phase contrast connected to a Leica TCS SP5 confocal imaging system (Leica, Wetzlar, Germany). Images were obtained using Leica LAS AF software and subsequently processed using Adobe Photoshop (Adobe, San Jose, CA, USA) and Volocity (Perkin Elmer, MA, USA). For live cell imaging, cells were incubated at 22 °C with directly conjugated probes. After washing, the cells were then transferred to an IbiTreat μ-slide 8-well microscopy chamber (Ibidi GmbH, Martinsried, Germany). The slides were imaged on the Leica SP5 confocal system using the environmental chamber for temperature control at 37 °C and CO_2_ enrichment.

### Comparative proteomics

Proteins in cell lysates were digested with trypsin, labeled with Tandem Mass Tag reagents according to the manufacturer's protocol (Thermo Fisher Scientific, Waltham, MA, USA) and combined in equal amounts. An aliquot of the pooled sample was evaporated to dryness and resuspended in buffer A (20 mM ammonium hydroxide, pH 10) prior to fractionation by high pH reversed-phase chromatography using an Ultimate 3000 liquid chromatography system (Thermo Fisher Scientific). In brief, the sample was loaded onto an XBridge BEH C18 Column (130 Å, 3.5 μm, 2.1 mm × 150 mm; Waters, Elstree, UK) in buffer A and peptides eluted with an increasing gradient of buffer B (20 mM ammonium hydroxide in acetonitrile, pH 10) from 0 to 95% over 60 min. The resulting fractions were evaporated to dryness and resuspended in 1% formic acid prior to analysis by nano-LC MS/MS using an Orbitrap Fusion Tribrid mass spectrometer (Thermo Scientific). Data acquisition and processing was performed as described previously^13^. For analysis, only rank 1 peptides and quantifications obtained using two or more unique peptides with high/medium confidence per protein were used. We selected a stringent comparative protein threshold of 2. Proteins that differed in level by twofold or more between replicate samples (due to heterogeneity between individuals) were excluded.

### Deformability measurements using ARCA

Packed red cells or retics were diluted 200-fold in a polyvinylpyrrolidone solution (PVP viscosity 28.1; Mechatronics Instruments, Netherlands). Samples of 200 μl were assessed in an Automated Rheoscope and Cell Analyzer (ARCA) consisting of a plate–plate optical shearing stage (model CSS450) mounted on a Linkam imaging station assembly and temperature controlled using Linksys32 software (Linkam Scientific Instruments, Surrey, UK). The microscope was equipped with an LMPlanFL × 50 with a 10.6 mm working distance objective (Olympus, Essex, UK) illuminated by a X-1500 stroboscope (Vision Light Tech, The Netherlands) through a band-pass interference filter (CWL 420 nm, FWHM 10 nm; Edmund Optics, Poppleton, UK). Images were acquired using an uEye camera (UI-2140SE-M-GL; IDS GmbH, Obersulm, Germany). At least 400 images per sample were analysed using bespoke ARCA software written by Dr J.G.G. Dobbe (Amsterdam Medical Centre, Netherlands).

### Hemox analysis

The oxygen carrying potential of the BEL-A cells, cultured adult erythroblasts and adult blood was determined using a hemox analyser (TCS Scientific Corp., New Hope PA, USA) according to the manufacturer's instructions at 37 °C. 10^8^ cells or 20 μl of pelleted BEL-A cells (differentiation day 13), cultured adult erythroblasts (day 14, 16% reticulocytes) or adult human PB drawn by lancet were diluted into 5 ml of HEMOX-Solution (TCS Scientific Corp.) containing 20 μl of Additive-A (BSA-20; TCS, Scientific Corp.) and 10 μl of anti-foaming agent (AFA-25; TCS, Scientific Corp.). The samples were oxygenated with air supplied from an external tank and deoxygenated with nitrogen gas. Changes in oxygen tension were measured using a Clark oxygen electrode. Increases in the oxyhaemoglobin fraction were simultaneously monitored by dual-wavelength spectrophotometry at 560 and 576 nm. The data were analysed using the Hemox Analytical Software (TCS, Scientific Corp.).

### *In vivo* studies

*In vivo* experiments were carried out in accordance with the Animals (Scientific Procedures) Act under licenses granted by the United Kingdom Home Office (PPL 30/3171). NSG mice were bred and maintained at the University of Bristol, Animal Service Unit. Adult mice were macrophage depleted by intravenous inoculation of liposome-encapsulated clodronate (dichloromethylene diphosphonate, CL2MDP, Clodronate Liposomes.com, the Netherlands) on day −3 (100 μl) and day −1 (50 μl). Mice were transfused with 1.7 × 10^8^ BEL-A cells or 2 × 10^8^ washed adult donor RBC via the left lateral tail vein. PB aspirates were taken from the right lateral tail vein at 10, 20, 40, 60, 120, 240, 480 min after inoculation and once daily thereafter for up to 9 days. Cells were stained with anti-human glycophorin A (BRIC256) conjugated to Alexa Fluor 488 and analysed by flow cytometry and confocal microscopy.

### Lentiviral transduction

For overexpression BEL-A cells and pro-erythroblasts differentiated from PB CD34^+^ cells were transduced with GPA-GFP, band 3-GFP and Lifeact-GFP in pXLG3 (ref. 34) in the presence of 8 μg ml^−1^ polybrene (Sigma-Aldrich), using identical cell numbers and virus batches. Transduction efficiency for each gene was measured 72 h post transduction with the same voltage for both cell types. For RhAG knockdown BEL-A cells were transduced with pLKO.1 short hairpin (sh) RNA plasmid TRCN0000083258 against RhAG (designed by the Broad Institute and purchased from Open Biosystems, GE Dharmacon, Lafayette, CO, USA), also in the presence of 8 μg ml^−1^ polybrene. After 24 h, the cells were washed with PBS and re-suspended in expansion medium containing puromycin (1 μg ml^−1^) for 48 h.

### Data availability

The data that support the findings of this study are available from the corresponding author upon reasonable request.

## Additional information

**How to cite this article:** Trakarnsanga, K. *et al*. An immortalized adult human erythroid line facilitates sustainable and scalable generation of functional red cells. *Nat. Commun.* **8,** 14750 doi: 10.1038/ncomms14750 (2017).

**Publisher's note**: Springer Nature remains neutral with regard to jurisdictional claims in published maps and institutional affiliations.

## Supplementary Material

Supplementary Information Supplementary Figures

Supplementary Data 1 Comparison of protein levels between BEL-A reticulocytes and reticulocytes from cultures of adult peripheral blood CD34+ cells. Proteins in cell lysates were digested with trypsin, labelled with Tandem Mass Tag (TMT) reagents and to analysed by nano-LC MS/MS. A comparative protein threshold of 2 was selected for differential expression. BEL-A1, 2, 3 and adult 1, 2, 3 are replicates, analysed in a 6-plex TMT analysis.

Supplementary Data 2 Proteins that differ in level between BEL-A reticulocytes and reticulocytes from cultures of adult peripheral blood CD34+ cells. (a) proteins more abundant in BEL-A reticulocytes than reticulocytes from cultures of adult peripheral blood CD34+ cells (b) proteins more abundant in reticulocytes from cultures of adult peripheral blood CD34+ cells than BEL-A reticulocytes.
