# Supplementary material for: An immortalized adult human erythroid line facilitates sustainable and scalable generation of functional red cells
Source: Nat Commun. 2017 Mar 14;8:14750. doi: 10.1038/ncomms14750 (PMC5355882; doi:10.1038/ncomms14750)
Supplement: Supplementary Information — Supplementary Figures [file ncomms14750-s1.pdf]

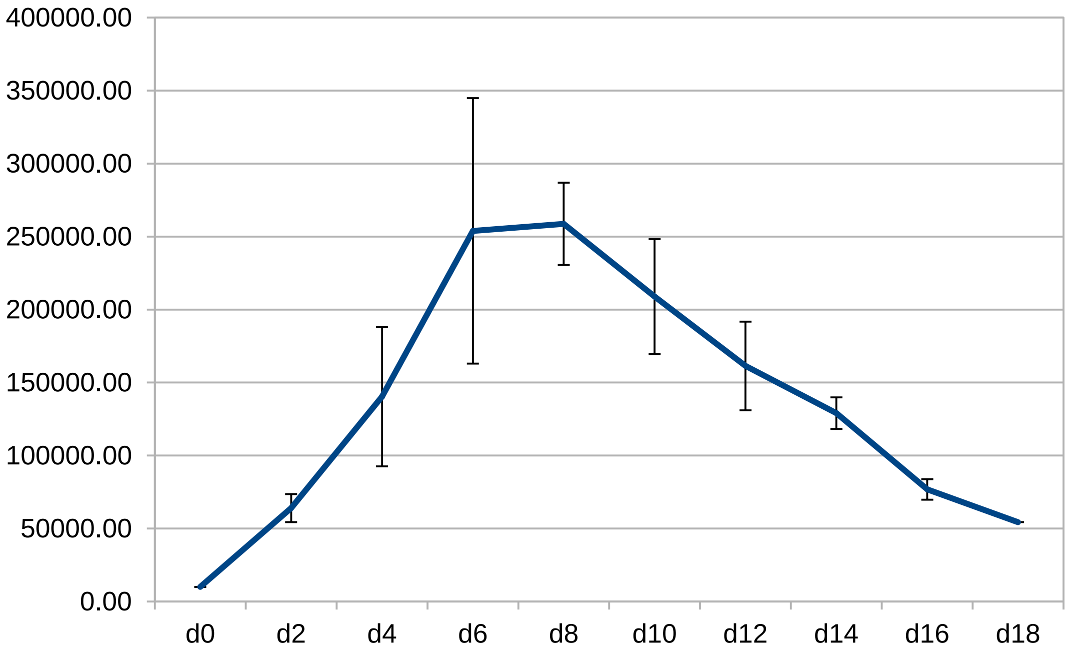

Supplementary Figure 1. Expansion profile of BEL-A cells following transfer to differentiation medium. Data shown are mean±s.d. n=3.

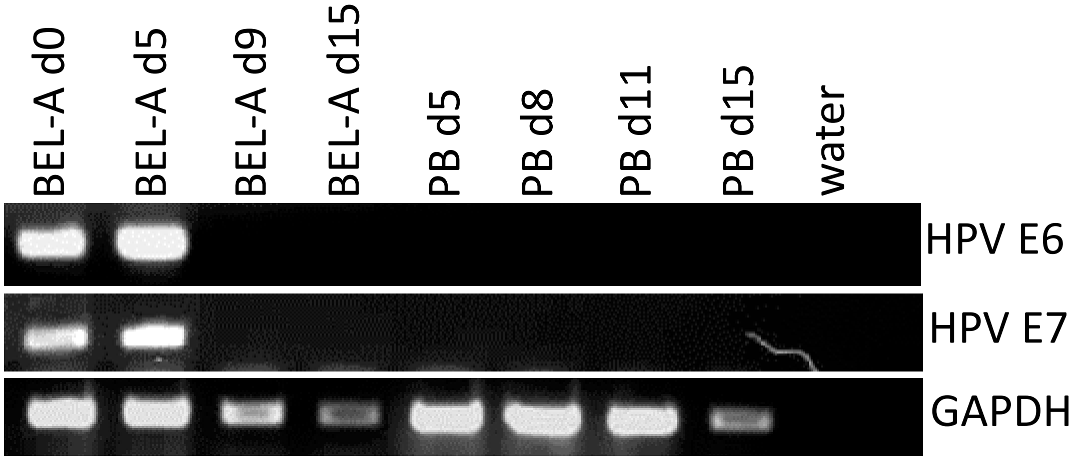

Supplementary Figure 2. Expression of HPV16 E6 and E7

BEL-A cells were maintained in expansion medium with doxycycline (day 0) before transfer to primary differentiation medium with doxycycline (day 1- 6) and tertiary differentiation medium (day 7 onwards). Erythroblasts differentiated from adult peripheral blood CD34^{+} cells were collected at day 5, 8, 11 and 15 in culture. Expression of HPV16 *E6* and *E7* was determined by the PCR. Expression of GAPDH was included as a positive control. The PCR reaction in lane ‘water’ did not contain cDNA.

Supplementary Fig. 3a

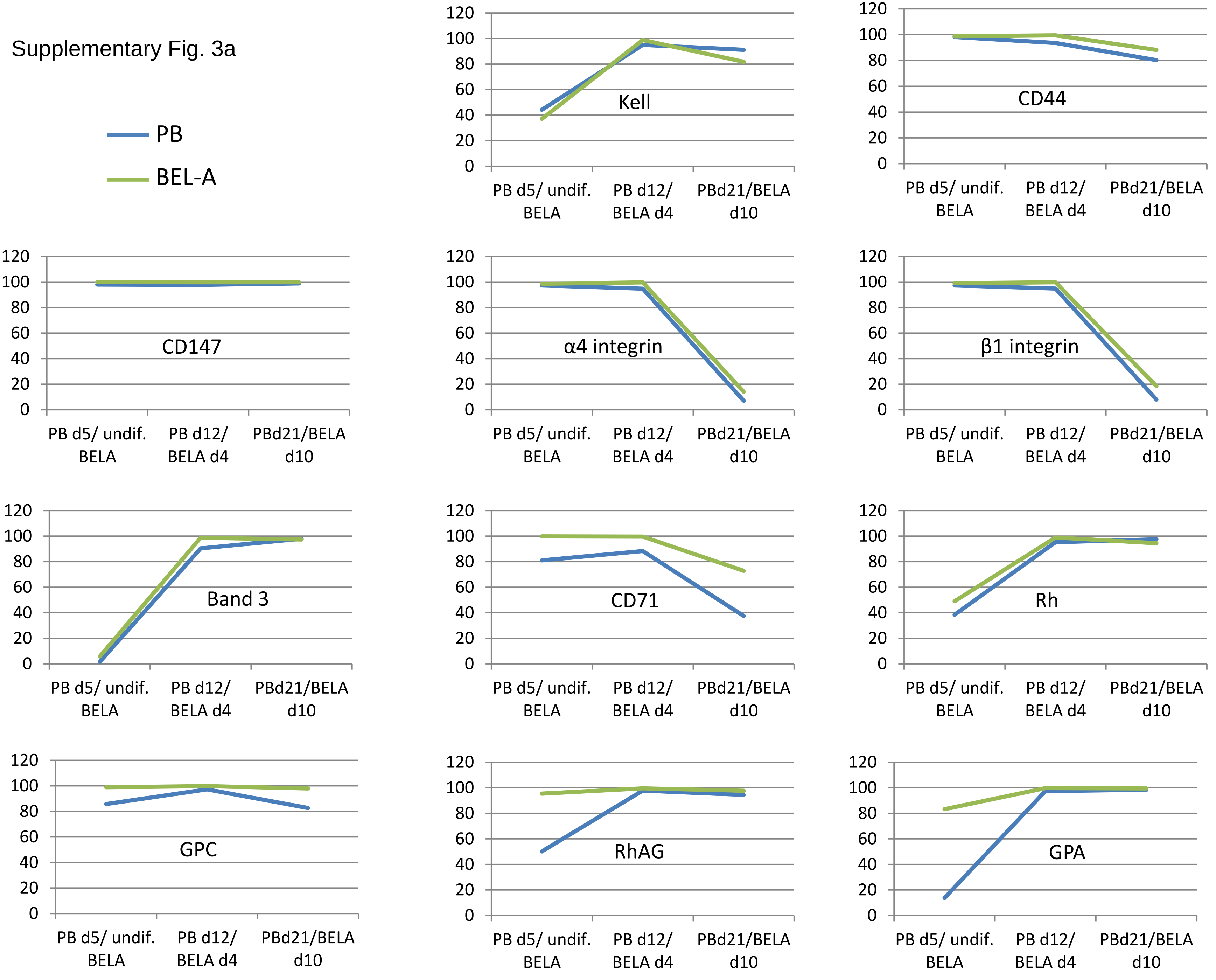

Supplementary Fig. 3b

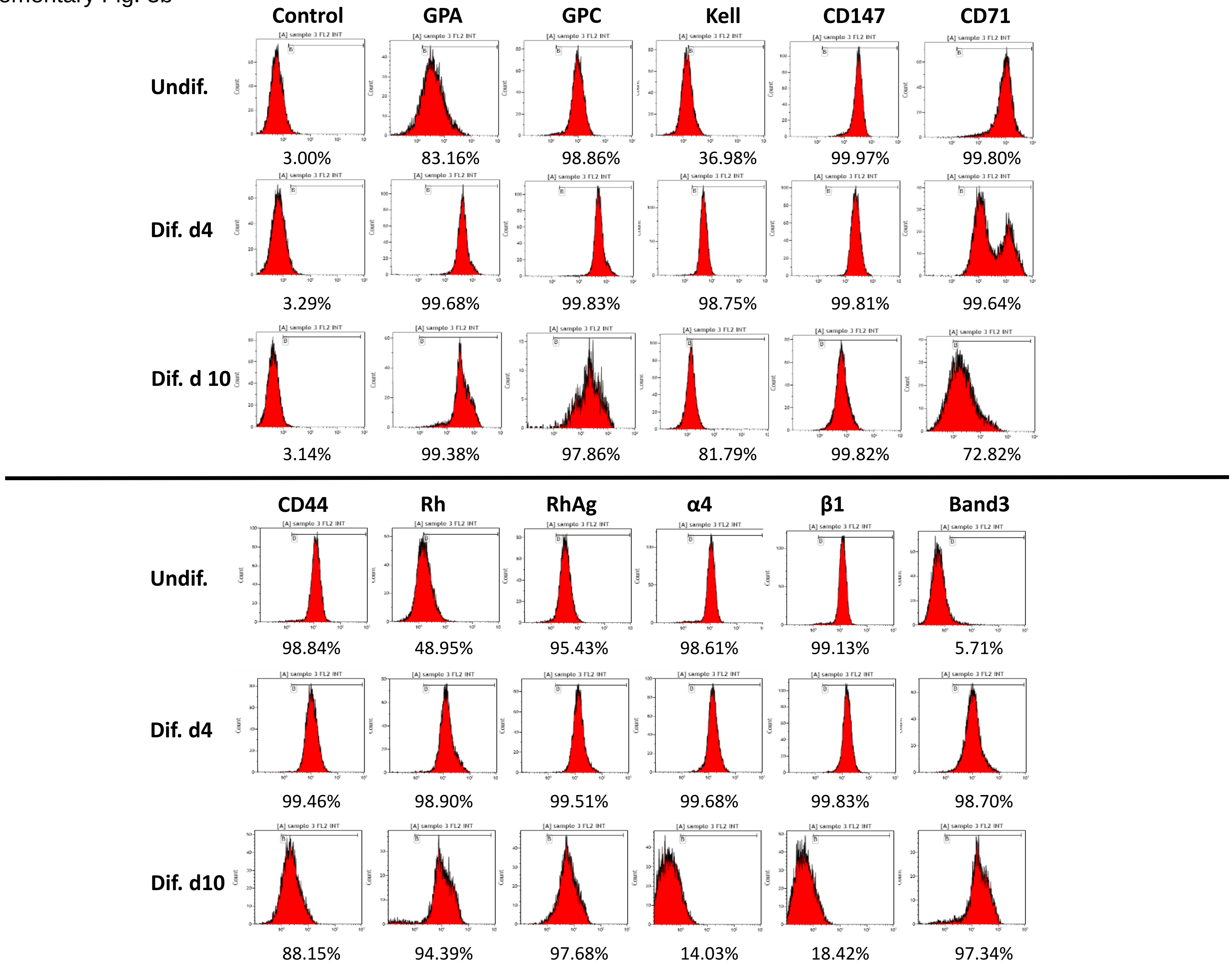

Supplementary Fig. 3c

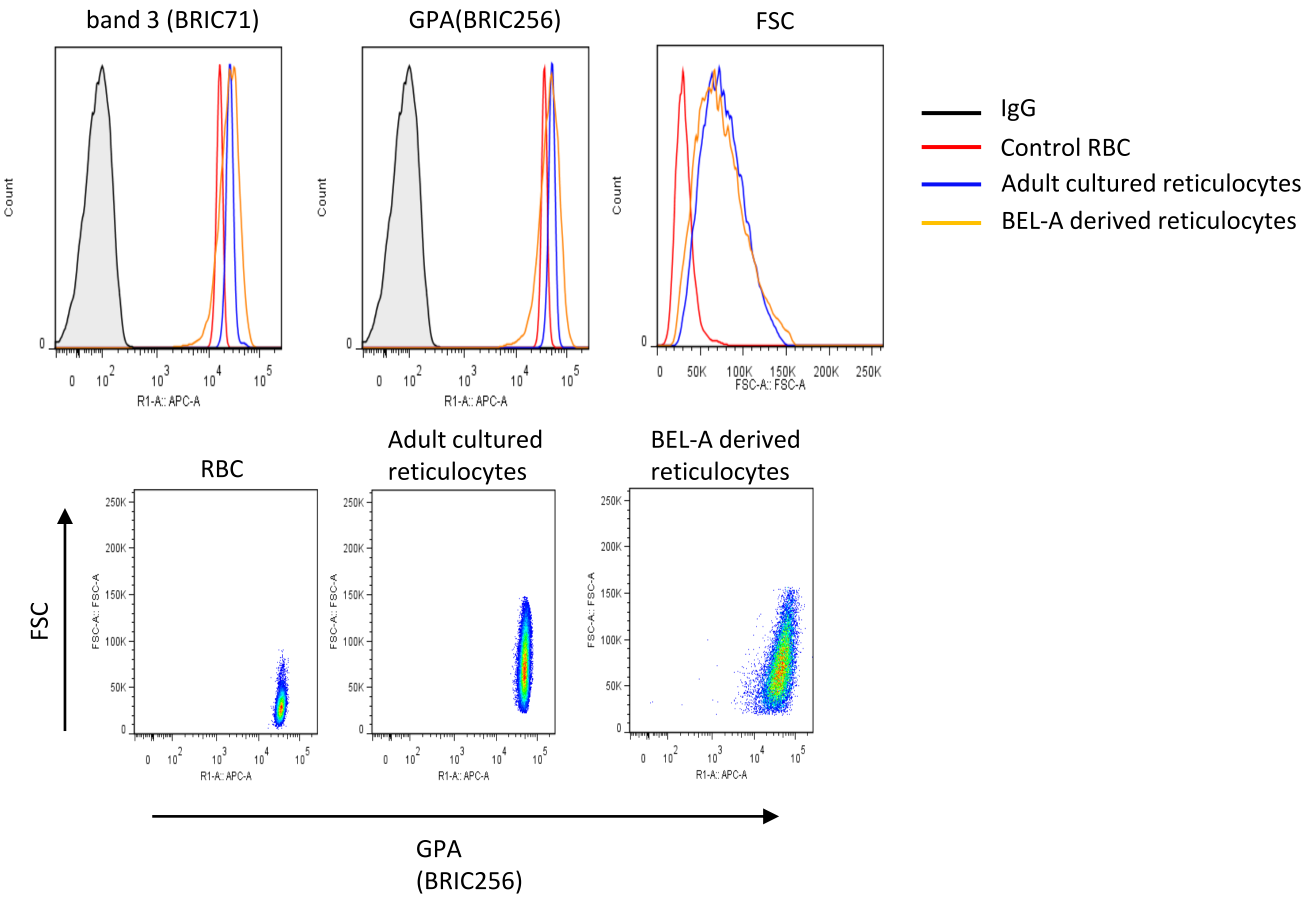

Supplementary Figure 3. Level of RBC membrane proteins in BEL-A compared to normal adult erythroid cells during erythropoiesis

BEL-A cells in expansion medium (undif), and at day 4 and 10 following transfer to differentiation media, and erythroblasts differentiated from adult peripheral blood $CD34^+$ cells at day 5, 12 and 21 in culture were incubated with antibodies to the proteins indicated and analysed by Flow Cytometry. (a) BEL-A cells compared to adult erythroid cells; y-axis indicates number of cell displaying fluorescent intensity (b) Histograms illustrating fluorescent intensity of BEL-A cells incubated with antibodies to indicated proteins at labelled stages of differentiation. Percentages refer to percentage positivity (c) Endogenous RBCs, BEL-A reticulocytes and *in vitro* adult reticulocytes were incubated with antibodies to glycophorin A (GPA) and band 3 (top row). Forward scatter (FSC) is also shown as an indicator of cell size. GPA expression again FSC is also shown (bottom row).

Supplementary Figure 4. Expression of key erythroid transcription factors in BEL-A compared to normal adult erythroid cells

RNA was isolated from BEL-A cells maintained in expansion medium and erythroblasts differentiated from adult peripheral blood $CD34^+$ cells at day 5 and 8 in culture. Expression of transcription factors was analysed by PCR. Expanding BEL-A cells are at a stage of differentiation closer to that of adult cells at day 5 than day 8 in culture.

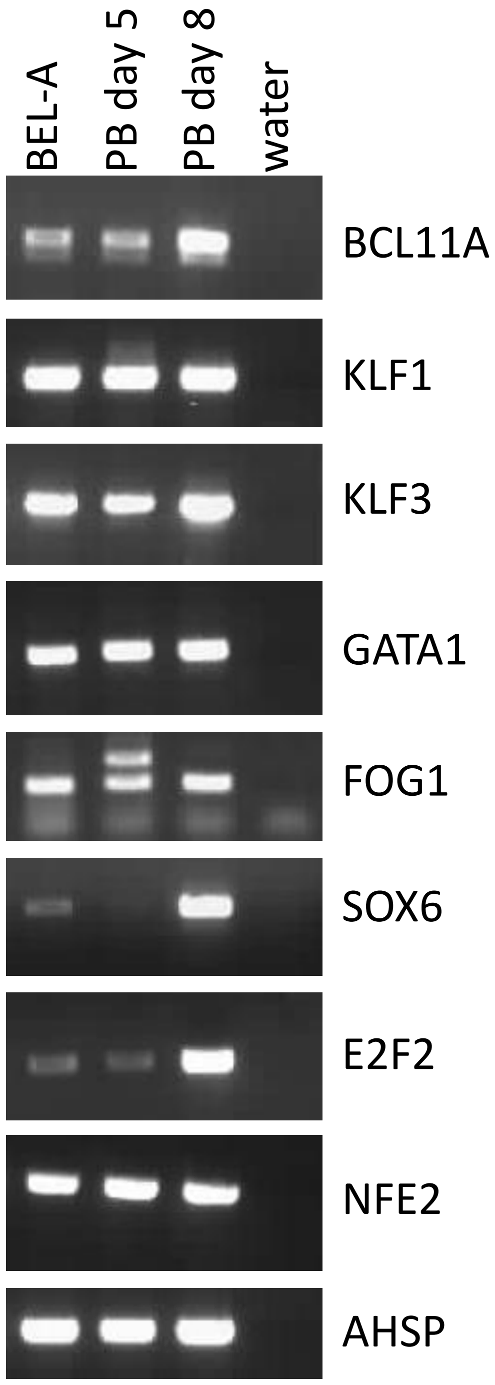

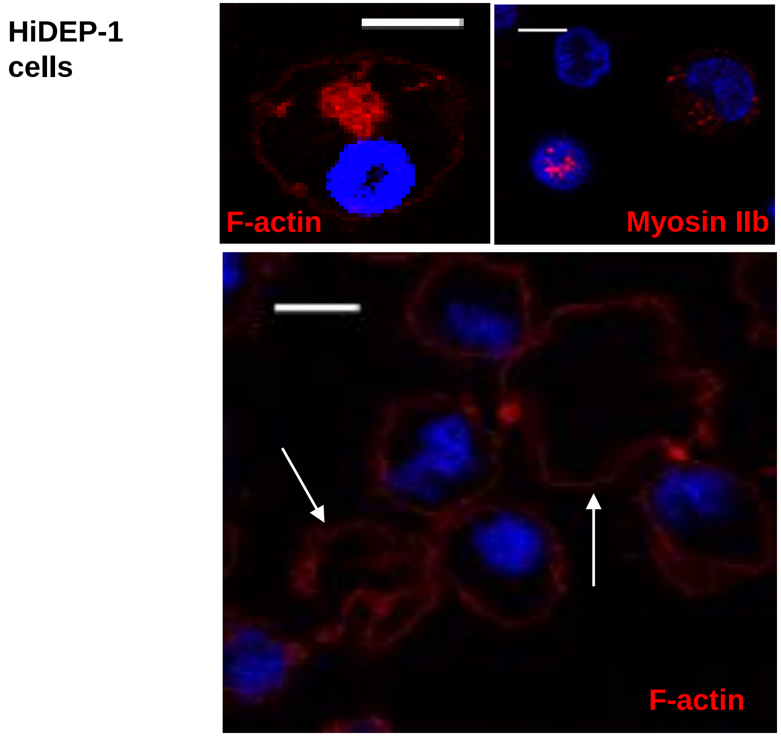

Supplementary Figure 5. Re-localisation of F-actin and myosin at enucleation in HiDEP-1 cells. Differentiating HiDEP-1 cells were fixed, permeabilsed and stained for F-actin and myosin IIb (red). Enucleating HiDEP-1 cells show cytoskeletal protein mis-localisation (upper panel). HiDEP-1 reticulocytes (white arrows) are morphologically abnormal (lower panel). Scale bars are 5um.

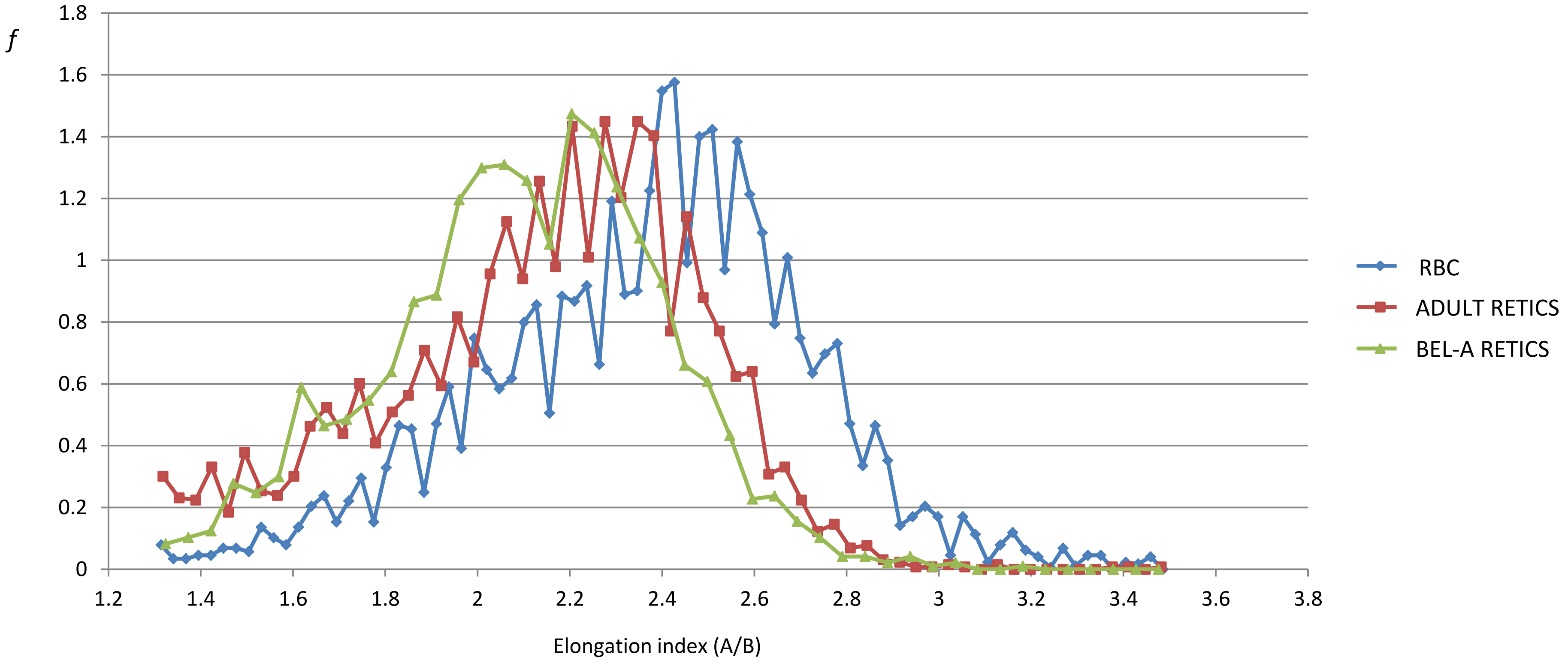

Supplementary Figure 6. Deformability of BEL-A reticulocytes

Adult reticulocytes (3-5 x10^6) differentiated from adult PB $CD34^+$ cells, BEL-A derived reticulocytes or donor red blood cells resuspended in 200µl PVP solution (viscosity at 37°C 28.1cP) were subjected to shear stress of 3Pa using an Automated Rheoscope Cell Analyser. At least 400 images per sample were analysed and the distribution of elongation indexes plotted in Microsoft Excel.

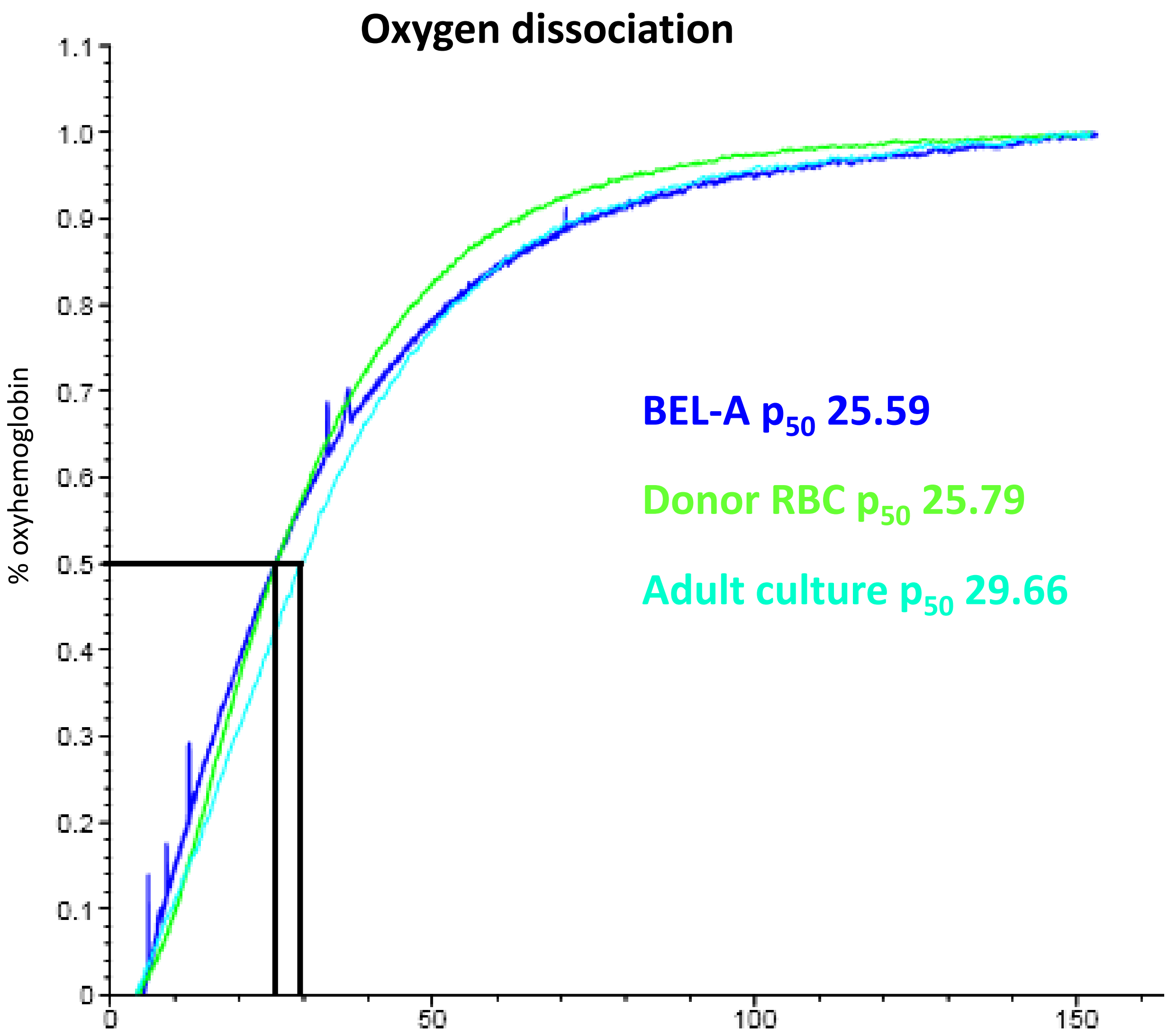

Supplementary Figure 7. Oxygen binding of BEL-A erythroid cells
The oxygen dissociation curve was plotted for 20µl of pelleted BEL-A cells (differentiation day 13), cultured adult erythroblasts (day 14, 16% reticulocytes) or adult human peripheral blood

$_{P}$O2

Supplementary Fig. 8

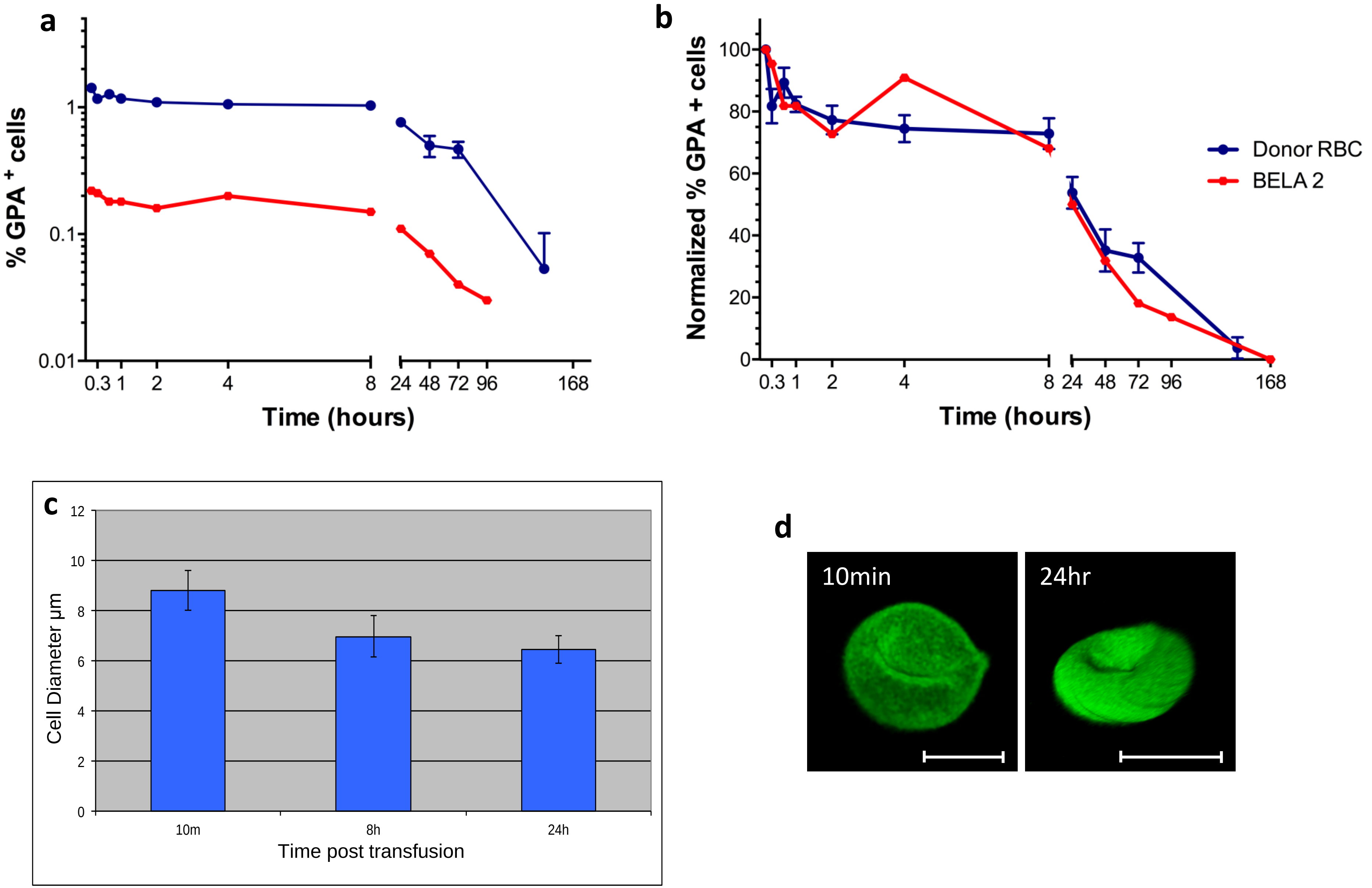

Supplementary Figure 8. Survival and maturation of BEL-A cells in macrophage depleted NSG mouse

BEL-A or adult donor RBCs were inoculated into the lateral tail vein of macrophage depleted NSG mice. Peripheral blood aspirates were taken from the opposite lateral tail vein at the time points indicated. Human cells were detected by expression of glycophorin A using flow cytometry (a) Percentage of human donor RBC or BEL-A cells in the mouse circulation (b) Proportion of human donor cells or BEL-A cells, normalized to 100% at 10 minutes after injection. Data represent mean±s.e.m. n=3 (c) The diameter of BEL-A cells measured at 10 minutes, 8 and 24 hours post transfusion Data represent mean±s.d. n=12 (d) 3D representation of BEL-A cells at 10 minutes and 24 hours post transfusion. Scale bars 5μm

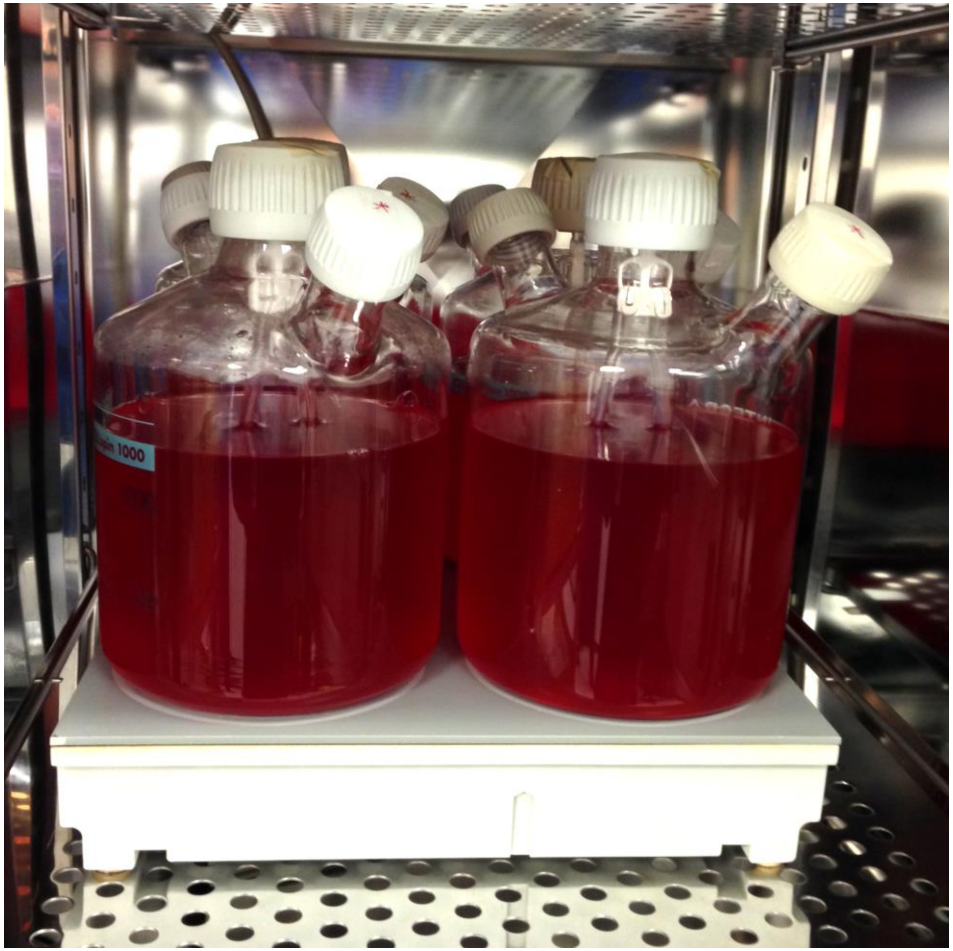

BEL-A cells in spinner flasks

Supplementary Figure 9. Scalability of the BEL-A line
BEL-A cells grown in static culture to 250ml were transferred to 1.5L spinner flasks. Expansion and differentiation was consistent to that of cells in static culture.

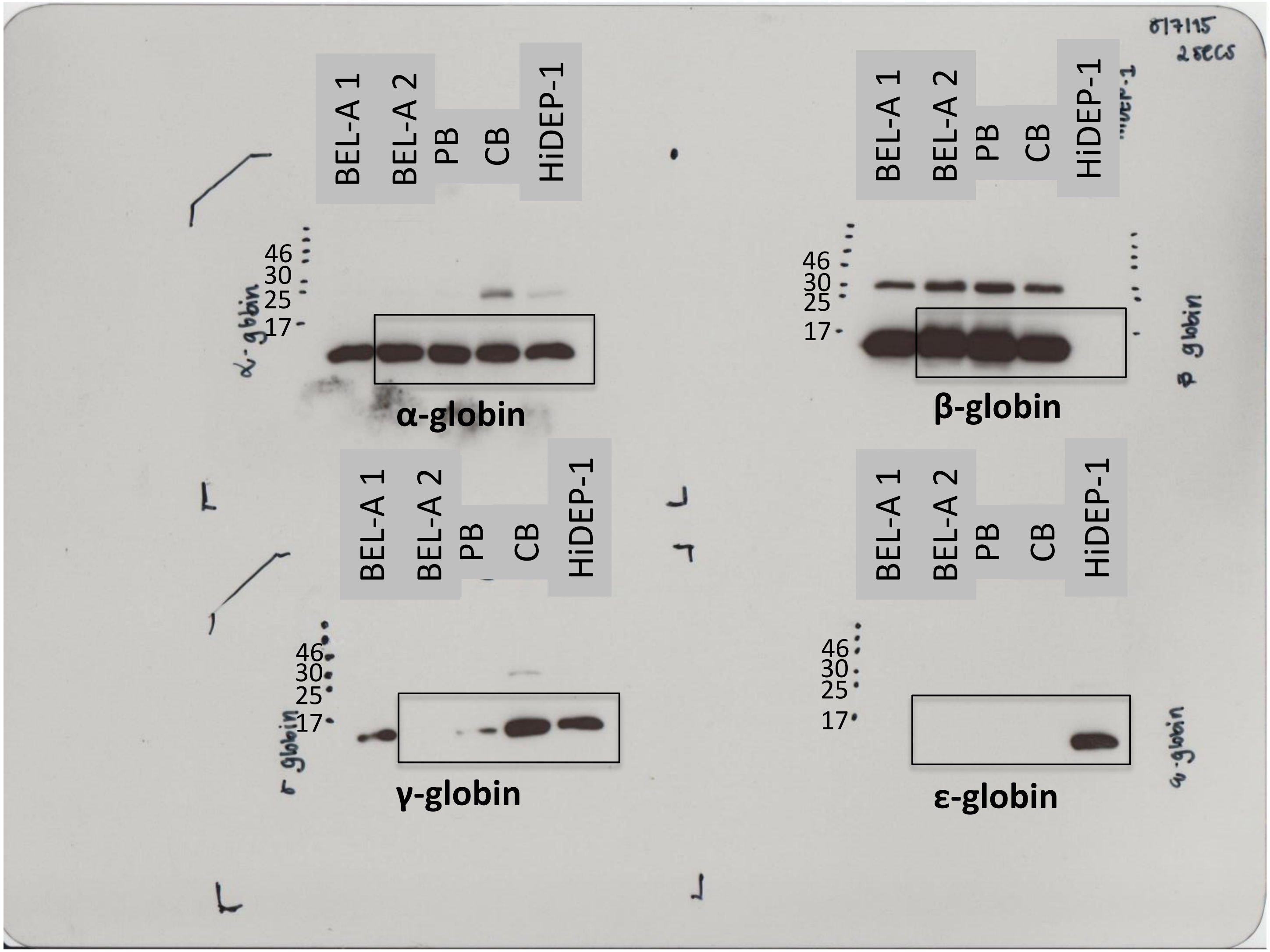

Supplementary Figure 10. Original film with full blots from which portions in boxed areas are shown in Figure 2a. The BEL-A cells described in the manuscript are in lane 2 of each blot (BEL-A 2). Globin expression in one of our additional immortalised adult erythroid lines is shown in lane 1 of each blot (BEL-A 1)

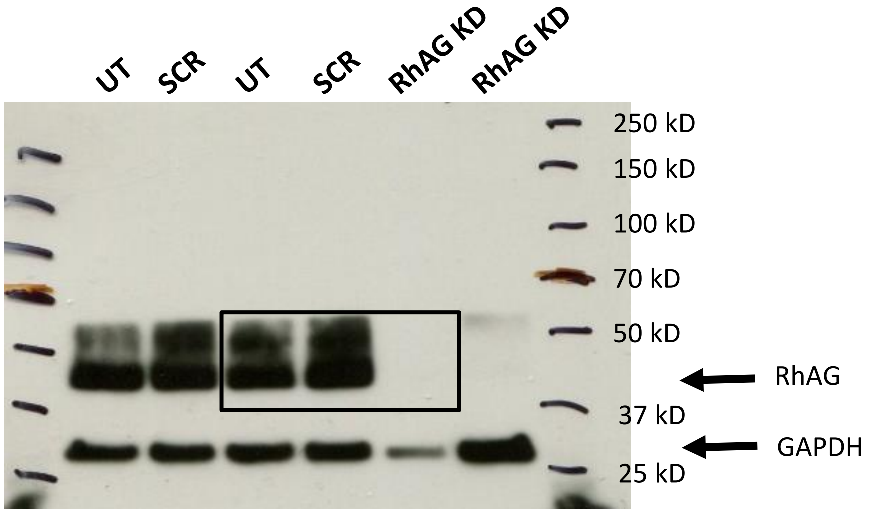

Supplementary Figure 11. Full blot from which portion in boxed area is shown in Figure 3. Membrane was also incubated with antibodies to GAPDH as a protein loading control
